# Increasing Incidence of Colorectal Cancer in Young Adults

**DOI:** 10.1155/2019/9841295

**Published:** 2019-11-11

**Authors:** Holli A. Loomans-Kropp, Asad Umar

**Affiliations:** ^1^Cancer Prevention Fellowship Program, Division of Cancer Prevention, National Cancer Institute, National Institutes of Health, 9609 Medical Center Dr., Bethesda, MD 20850, USA; ^2^Gastrointestinal and Other Cancers Branch, Division of Cancer Prevention, National Cancer Institute, National Institutes of Health, 9609 Medical Center Dr., Bethesda, MD 20850, USA

## Abstract

**Background:**

Colorectal cancer (CRC) incidence and mortality has been declining in the U.S. Despite success in reducing CRC incidence, incidence of early-onset CRC has increased markedly. In this study, we identified age-related disparities in CRC incidence and mortality, and investigated differences in anatomical distribution of colon cancers between populations.

**Methods:**

CRC trends were evaluated using Surveillance, Epidemiology, and End Results Program Data from 1980–2016 for individuals under age 50 and 50 years and older. Rates and ratios were calculated using SEER^∗^Stat. Regression analyses were calculated using Joinpoint.

**Results:**

Increased CRC incidence among individuals under age 50 was observed. Among individuals under age 50, incidence-based mortality (IBM) stabilized, while incidence and IBM decreased for individuals aged 50 years and older. Normalized trends indicated increased rectal cancer incidence for individuals under age 50, particularly among individuals aged 30–39. Similar incidence of proximal and distal colon cancers in individuals under age 50 was observed, while colon cancers in individuals aged 50 and older were primarily distal.

**Conclusions:**

We found age-related disparities in CRC incidence and IBM between individuals under age 50 and age 50 years and older. Increasing incidence rates of rectal cancer substantially accounts for this disparity among individuals under age 50. The escalating trends of early-onset CRC warrant investigation into the factors leading to the population-level trends.

## 1. Introduction

Colorectal cancer (CRC) incidence and mortality has been declining in the United States for over a decade. Despite the decreasing trend, CRC remains the third most incident and fatal cancer. In 2019, CRC will be responsible for an estimated 78,500 new cases in men and 67,100 new cases in women, an increase of approximately 3.8% from 2018 for men and women[1, 2]. Fortunately, there are well-established screening guidelines that allow for the prevention and early detection of CRC [3–5]. Despite the recommendations issued by the United States Preventive Services Task Force (USPSTF) and the American Cancer Society, adherence to these guidelines remains low despite national efforts to improve screening rates [6–8].

Existing CRC screening guidelines do not sufficiently address a newly emerging high-risk group: early-onset colorectal cancers (EOCRCs). EOCRC is defined as a cancer diagnosis occurring in individuals under the current recommended screening age of 50 [8]. Though substantial efforts have been directed towards the identification and understanding of the underlying genetics of hereditary CRC and recommendations are in place to screen individuals with a familial history of the disease, those presenting with sporadic CRC are typically missed by screening [9, 10]. Recent estimates indicate that sporadic CRC accounts for approximately 70% of EOCRCs [11]. Studies have shown that EOCRCs have increased by 2.8%–36.5% globally over the last several decades; however, this is not suggestive of increasing incidence of hereditary CRCs [12–14]. Siegel and colleagues investigated this phenomenon using Surveillance, Epidemiology, and End Results (SEER) Program Data and found that CRC rates have been increasing annually in individuals aged 20–39 and 40–54 since the mid-1980s and mid-1990s, respectively [15]. Several recent investigations have demonstrated similar trends throughout Europe, Australia, and New Zealand [16, 17]. Though not as drastic, similar trends have been observed for mortality in these age groups [16, 18]. This lack of screening in the early-onset population often results in delayed diagnoses and patients presenting with symptoms and late stage, poorly differentiated cancers [19–22].

In recent years, alterations in diet, sedentary lifestyles, and the rising prevalence of obesity have been hypothesized to impact molecular and physiological characteristics influencing the risk of CRCs and other cancer types in younger populations [23–26]. Additional insights into the clinicopathology and speculated etiology and risk factors of EOCRC have been comprehensively reviewed [27–29]. Several recent studies have sought to further delineate the pathology and genetics of EOCRC, however these studies are limited by issues such as small sample size, among others [30–33]. Due to the observed age-related disparities and hypothesized differences in etiology based on environmental and lifestyle factors in CRC development, we sought to investigate palpable differences between early-onset and representative CRCs using population-based data. Our study provides the most recent quantitative assessment of age-related disparities in CRC incidence and mortality rates in cases using the current release of SEER Program Data, from 1980 to 2016.

## 2. Materials and Methods

### 2.1. Study Design

To investigate changes in CRC trends in the United States over time, we performed a retrospective analysis of patients aged 20–49 and 50 and older diagnosed with colon and rectal cancers from 1980–2016 using SEER Program Data. The SEER registry is a population-based, comprehensive deidentified database of annual cancer incidence and mortality in the United States. Data collected within SEER represents approximately 28% of the national population. For this analysis, we used data collected from the nine oldest SEER catchment areas (SEER9: Atlanta, Connecticut, Detroit, Hawaii, Iowa, New Mexico, San Francisco-Oakland, Seattle-Puget Sound, and Utah) and the most recent expansion to 18 catchment areas (SEER18: SEER9 areas, Los Angeles, San Jose-Monterey, Rural Georgia, the Alaskan Native Tumor Registry, Greater California, Greater Georgia, Kentucky, Louisiana, and New Jersey). Cases were identified within the program data using ICD-O-3 codes (colon: C18.0, C18.2–C18.9, C26.0; rectum: C19.9, C20.9). To investigate differences in incidence and mortality by colon cancer anatomical subsite, we stratified cases by ICD-O-3 codes corresponding to proximal and distal colon cancers (proximal colon: C18.0, C18.2–C18.4; distal colon: C18.5–C18.7). Codes identifying colon cancer lesions with an unspecified origin or of overlapping subsite were excluded from the analysis of proximal and distal colon cancer rates.

Mortality data were collected by the National Center for Health Statistics (NCHS). Only deaths with the underlying cause of colon or rectal cancers with the specified ICD-O-3 codes were included in the analysis. Due to limited sample size and to strengthen our analyses, CRC trends were not stratified by race. As the data used to conduct this study were publicly available and deidentified, review and approval by the institutional review board was not required.

### 2.2. Statistical Analysis

Data access and analysis were performed using SEER^∗^Stat (version 8.3.5, National Cancer Institute [NCI]). Within SEER^∗^Stat, CRC incidence calculations were delay-adjusted. Incidence rates were calculated as the number of cases or deaths per 100,000 persons and age-adjusted to the 2000 U.S. standard population. Incidence-based mortality (IBM) allows for the ability to subdivide mortality by variables associated with the onset of cancer [34]. CRC incidence and IBM rates were calculated for grouped (under age 50, 50 and older) and for seven separate age groups (20–29, 30–39, 40–49, 50–59, 60–69, 70–74, 75+). To perform additional analyses, we collapsed age groups (20–49, 50 and older) to allow for sufficient numbers in the calculation of incidence and mortality rates by anatomical subsite. Data were graphed as age-adjusted rates per 100,000 persons or as normalized age-adjusted rates per 100,000 persons, using the 1980 age-adjusted rate as baseline.

Temporal trends were calculated using the Joinpoint Regression Program (version 4.5.0.1, NCI), which uses Monte Carlo permutation to fit a series of linear joinpoints to the provided data and estimate annual percent change (APC) [35]. A maximum of four joinpoints were calculated per analysis. The reported APCs were considered statistically significant when the value was different from zero, determined using a two-sided test. All additional statistical tests were two-sided. A *p*-value less than 0.05 was considered statistically significant. Graphs were visualized using Prism (GraphPad, version 7.01).

## 3. Results

**Figure 1:**
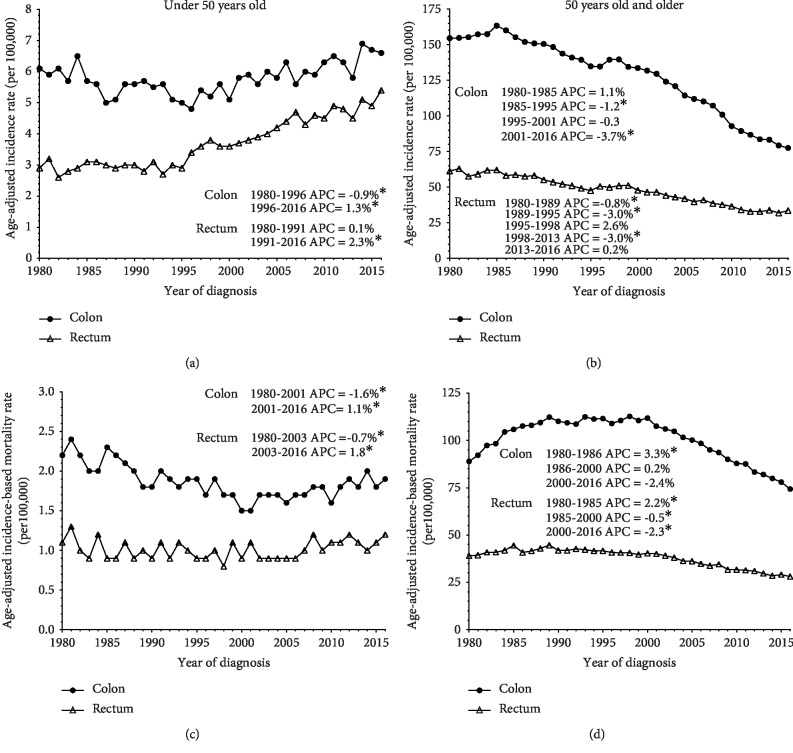
Colon and rectal cancer incidence and mortality rates stratified by age, 1980–2016. Age-adjusted incidence and incidence-based mortality (IBM) rates per 100,000 persons from 1980–2016 were derived from SEER9 program data. Colon and rectal cancer incidence and IBM trends of individuals under the age of 50 (a, c) and age 50 and older (b, d). Annual percentage change (APC) are quantifications of the changes in trends. Asterisks (^∗^) designate statistical significance calculated in the permutation model.

Between 1980 and 2016, 37,138 and 433,012 incident colorectal cancer cases were recorded in individuals aged 20–49 and 50 and older, respectively, in SEER9. Of these cases, 313,375 (72.4%) were colon cancers and 119,637 (27.6%) were rectal cancers in individuals aged 50 and older, while 22,425 (60.4%) were colon cancers and 14,713 (39.6%) were rectal cancers among individuals under the age of 50. Though the number of cases of colon and rectal cancers for individuals aged 50 and older far exceeded those diagnosed in individuals aged 20–49, the incidence rate trends between the two groups differed. Among those under the age of 50, age-adjusted incidence rates for colon cancer declined by 0.9% annually (95% confidence interval (CI): −1.6%, 0.2%) between 1980 and 1996, however rates increased by 1.3% annually (95% CI: 0.9%, 1.7%) between 1996 and 2016 (Figure 1(a)). Rectal cancer incidence rates have significantly increased by 2.3% annually since 1991 (95% CI: 2.1, 2.6) in this group. Individuals aged 50 and older demonstrated opposite trends, showing relatively steady declines in age-adjusted incidence for colon and rectal cancers since 2001 and 1998, respectively (Figure 1(b)). Similar trends in IBM rates were observed in both age groups (Figures 1(c) and 1(d)).

**Figure 2:**
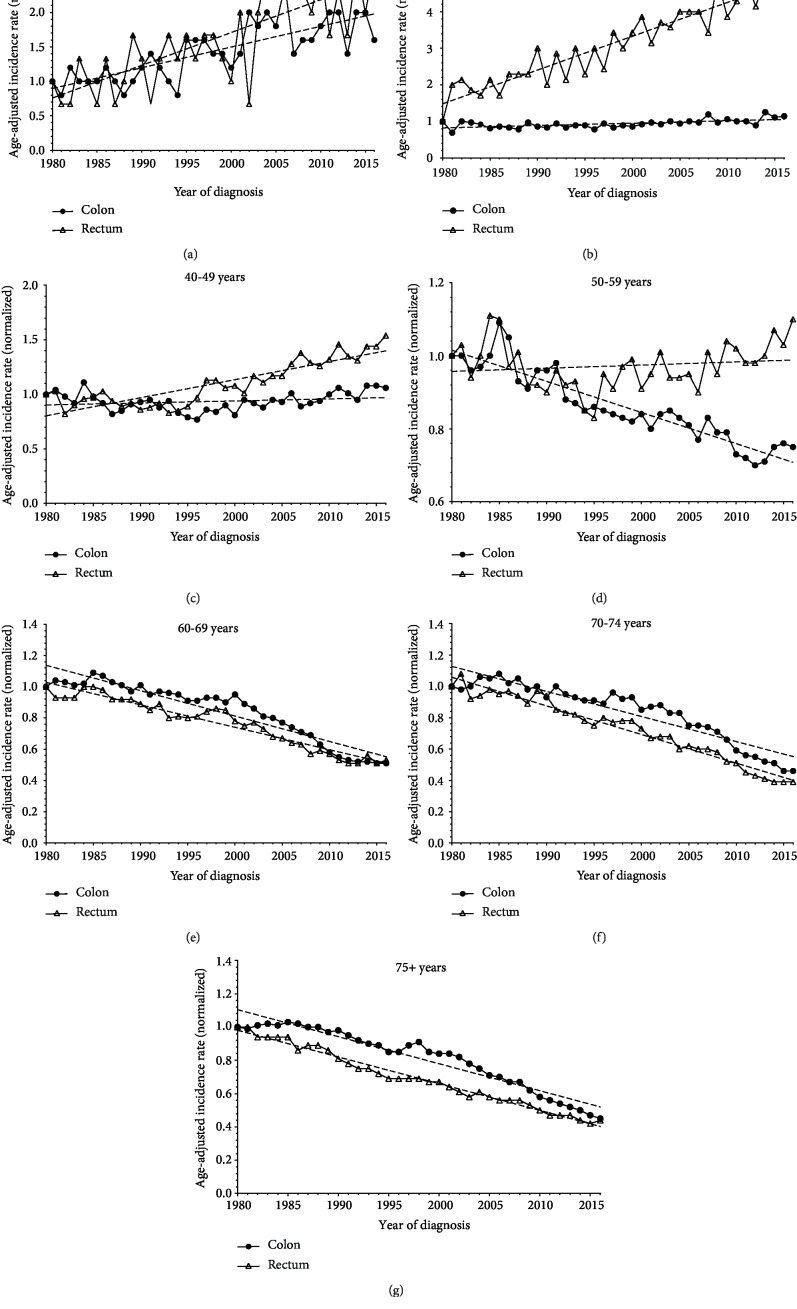
Comparison of normalized incidence and mortality rates between age 20–49 and 50 and older by tumor subsite, 1980–2016.****Rates of individuals age 20–49 and 50 and older from 1980–2016 from SEER9 were normalized to the 1980 rate to better visualize the magnitude of differences in incidence and IBM trends by age over time. Normalized rates were divided into 10-year age intervals: 20–29 (a), 30–39 (b), 40–49 (c), 50–59 (d), 60–69 (e), 70–74 (f), and 75+ (g).

The disparity in colon and rectal cancer trends for individuals aged 20–49 and 50 and older may be difficult to appreciate, as the absolute number of colon and rectal cancer cases per stratified age group differ drastically. Among individuals aged 20–29, normalized colon and rectal cancer incidence rates increased from 1980 to 2016 (Figure 2(a)). Interestingly, for individuals aged 30–39 and 40–49, only rectal cancer incidence demonstrated consistent increasing trends over the studied period (Figures 2(b) and 2(c)). Rectal cancer incidence trends steadily increased after 2008 among individuals aged 50–59 (Figure 2(d)). Colon cancer rates remained stagnant over time among individuals aged 30–39; however, a slight increase in trends was noted for individuals aged 40–49 in 2003 (Figures 2(b) and 2(c)). Decreasing colon and rectal cancer incidence was consistent over time for individuals aged 50 and older (Figures 2(d)–2(g)). The slope of incidence rate decline was similar for colon and rectal cancer for individuals aged 60–69; however, the rate of decline for rectal cancer incidence was greater for individuals aged 70–74 and 75+ over time, compared to colon cancer (Figures 2(e)–2(g)).

**Figure 3:**
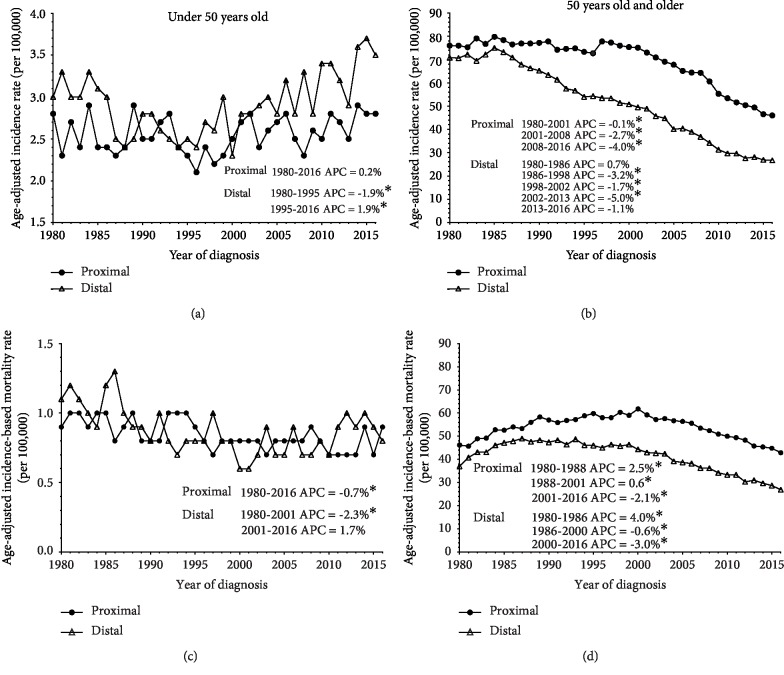
Trends in colon tumor subsite-specific incidence and mortality rates among individuals age 20–49 and 50 and older, 1980–2016.****Age-adjusted incidence and mortality rates per 100,000 from 1980–2016 were derived from SEER9 program data and subdivided into proximal and distal colon tumor subsites using ICD-O-3 codes, to examine rates over time. Proximal and distal colon cancer incidence and IBM rates are depicted for age 20–49 (a, c) and 50 and older (b, d). Asterisks (^∗^) designate statistical significance calculated in the permutation model.

As approximately 60% of diagnosed CRCs arise in the colon, as previously noted [15]. Therefore, we wanted to investigate identifiable intrinsic differences in colon cancer incidence and mortality between age groups. Previous studies have indicated that distal colon tumors are more commonly diagnosed than proximal tumors in early-onset colon cancer, particularly tumors located in the cecum and sigmoid colon; proximal colon tumors occur more frequently in older individuals [21, 36, 37]. Our analysis indicated a similar pattern. Individuals under the age of 50 had an overall higher incidence of distal colon cancers compared to proximal tumors, though the magnitude of difference between the two groups was not large. Interestingly, a significant increase in distal colon cancers was observed from 1995 to 2016 (APC = 1.9%, 95% CI: 1.4%, 2.4%), while this trend was not observed for proximal cancers (APC = 0.2%, 95% CI: −0.1%, 0.5%) in this group (Figure 3(a)). In contrast, substantial differences were observed in colon cancer-specific subsites for individuals aged 50 and older. From 1980 to 2016, proximal colon cancers had higher incidence rates compared to distal colon cancers though the magnitude varied considerably over time, ranging from a minimum rate difference of 3.1 per 100,000 persons to a maximum rate difference of 27.7 per 100,000 persons (Figure 3(b)). A comparison of mortality rates of individuals under the age of 50 to individuals aged 50 and older revealed that both groups had higher IBM rates for proximal colon cancers than distal colon cancers (Figures 3(c) and 3(d)). However, among individuals under the age of 50, the IBM rates between proximal and distal colon cancers began equalizing in 2003, while the difference in mortality rates between proximal and distal cancer became more pronounced for individuals aged 50 and older. IBM rates for distal colon cancer among individuals under the age of 50 decreased significantly from 1980 to 2001 (APC = −2.3%, 95% CI: −3.2%, −1.4%); however, a nonsignificant 1.7% APC (95% CI: 0.0%, 3.5%) from 2001 to 2016 was noted (Figure 3(c)). Among individuals aged 50 and older, IBM significantly decreased for both proximal and distal colon cancers since 2001 and 1986, respectively (Figure 3(d)).

**Figure 4:**
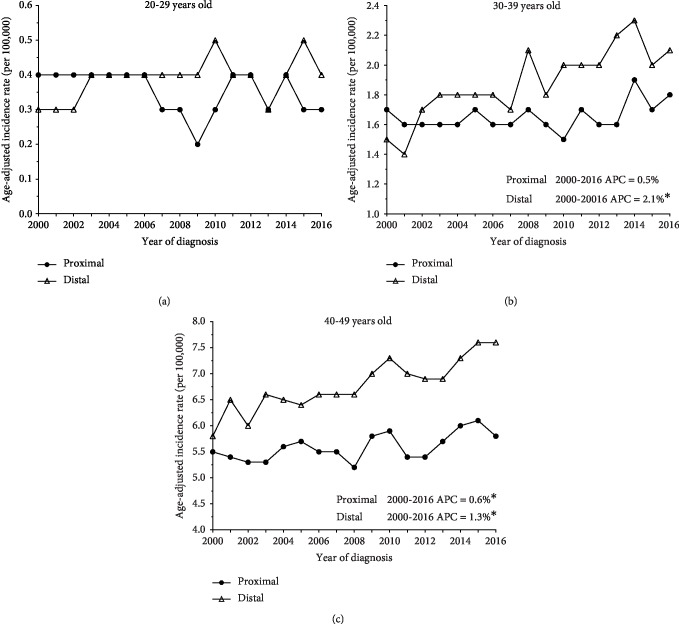
Trends in colon tumor subsite-specific incidence rates stratified by age, 2000–2016.****Age-adjusted incidence rates per 100,000 persons from 2000–2016 were derived from SEER18 program data. Colon cancer incidence trends were calculated for individuals age 20–29 (a), 30–39 (b), and 40–49 (c). Annual percentage change (APC) are quantifications of the changes in trends. Asterisks (^∗^) designate statistical significance calculated in the permutation model.

Because of the intriguing variation and increasing trends in proximal and distal colon cancer incidence among individuals under the age of 50, we decided to further investigate these trends in SEER18 program data and stratified age groups (20–29, 30–39, 40–49). When we stratified by age, we found that age-adjusted incidence rates of proximal and distal colon cancers were similar in individuals aged 20–29 years (Figure 4(a)). Due to low incidence rates, APC could not be calculated for this age group. Incidence rate ratios (IRR) for proximal and distal colon cancers for individuals aged 20–29 did not significantly differ between 2000 and 2014, using the year 2000 as reference (Supplemental [Supplementary-material supplementary-material-1]). Distal colon cancer, however, exhibited an increased IRR in 2015 (IRR = 1.57, 95% CI: 1.02, 2.44). Individuals aged 30–39 and 40–49 had higher incidence rates for distal colon cancer than proximal colon cancer, showing a 2.4% (95% CI: 1.6%, 3.3%) and 1.2% (95% CI: 0.8%, 1.6%) APC in distal colon cancer incidence since 2000 (Figures 4(b) and 4(c)). Like that observed in individuals aged 20–29, individuals aged 30–39 and 40–49 had increased IRRs for distal colon cancers between 2008 and 2016 and 2006 and 2016, respectively (Supplemental [Supplementary-material supplementary-material-1]). Proximal colon cancers did not express this pattern.

Collectively, these results indicate an age-related shift in both colon and rectal cancers, though the increasing incidence rates of rectal cancers is more pronounced among individuals under the age of 50. We additionally found that, when colon cancers were further examined, incidence rates of distal cancers increased among those under the age of 50.

## 4. Discussion

The work presented here illustrates the magnitude of increased EOCRC incidence from 1980 to 2016. A corresponding decrease in CRC was observed for individuals aged 50 and older in incidence and IBM. We additionally demonstrated the increasing rate of distal colon cancers among individuals under the age of 50. Individuals aged 50 and older were primarily diagnosed with proximal colon cancers, though incidence and IBM decreased over time for both proximal and distal cancers in this age group. Though CRC incidence of those aged 50 and older is higher than those under 50, the rate of change observed for individuals under age 50 far exceeds that of individuals aged 50 and older. The data presented here additionally validates investigations reported globally [16, 17]. The disparities in presentation reported here may reveal intrinsic differences (e.g., genetic, molecular, and pathological) in colon and rectal cancers across age groups. Therefore, we should now seek to improve overall outcomes for EOCRC by becoming vigilant in diagnosing the disease at time of presentation, particularly as early onset cases usually have a longer duration of symptoms, time to diagnosis, and time to evaluation [20, 38].

There are, however, obstacles impacting the ability to combat increasing EOCRC. Salimzadeh and colleagues assessed knowledge of individuals with first-degree relatives (FDRs) with colon cancer and found no difference in knowledge of colon cancer risk between FDRs and individuals at moderate risk, indicating a general lack of colorectal cancer risk awareness [39]. Dissemination of screening information and targeted messaging for colon and rectal cancers has the potential to increase population awareness of the signs and symptoms of the disease, screening practices, and recommended guidelines.

Studies have recently emerged suggesting lowering the recommended age to begin colorectal cancer screening. Microsimulation models, which account for variables such as age, gender, screening modality, and interval of screen, are often used to assess the cost-benefit analysis of different screening strategies. Current models of CRC screening have suggested benefit, measured primarily by life-year gain, beginning CRC screening at age 45 [40, 41]. In fact, the American Cancer Society published an update to their guidelines, lowering the recommended screening age to 45, based on the results of the microsimulation data [5]. Current guidelines already emphasize CRC screening at a younger age for particular subsets, such as African Americans [4]. Therefore, the aforementioned guideline revision and the results of the current study highlight the essential need to address the rising rates of EOCRC at both the individual- and population-levels.

### 4.1. Limitations

The current study was conducted using SEER program data, a rich resource used to examine cancer trends in the United States over time. However, because this is a national, publicly available resource, there are limitations to the use of the data. For example, SEER data allow for the retrospective analysis of cancer trends, with a lag time in data release. We are then only able to examine trends over time and not able to glean other associations and causality. Additionally, SEER program data rely on complete records to include ICD codes and histology, which may not be available for all patients and does not account for hereditary versus sporadic causes of cancer. This can be especially problematic for EOCRC, as the number of cases available may be limited.

### 4.2. Conclusion

Despite progress in reducing overall colorectal cancer incidence and mortality in the United States, an opposite alarming trend continues among younger individuals. This age group is a key demographic in which screening or cancer preventive efforts are not recommended. The data presented here, however, suggest that additional research is warranted to reduce this age-related disparity. The escalating trends of EOCRC support further investigation into the lifestyle, behavioral, environmental, and genetic factors, or the synergistic action of the different factors, that are responsible for the increasing population-level trends.

## Data Availability

All data used in the study are publicly available from the Surveillance, Epidemiology, and End Results Program (SEER; https://seer.cancer.gov) of the National Cancer Institute.

## Abbreviations

APC: Annual percent change
CRC: Colorectal cancer
EOCRC: Early-onset colorectal cancer
IBM: Incidence-based mortality
ICD: International classification of diseases
NCHS: National Center for Health Statistics
SEER: Surveillance, Epidemiology, and End Results
USPSTF: United States Preventive Services Task Force.

## Supplementary Materials

Supplementary Materials Incidence rate ratios (IRR) and 95% confidence intervals (95% CI) for proximal and distal colon cancers, stratified by age.
