# Supplementary figures and images for: Increasing Incidence of Colorectal Cancer in Young Adults
Source: J Cancer Epidemiol. 2019 Nov 11;2019:9841295. doi: 10.1155/2019/9841295 (PMC6885269; doi:10.1155/2019/9841295)

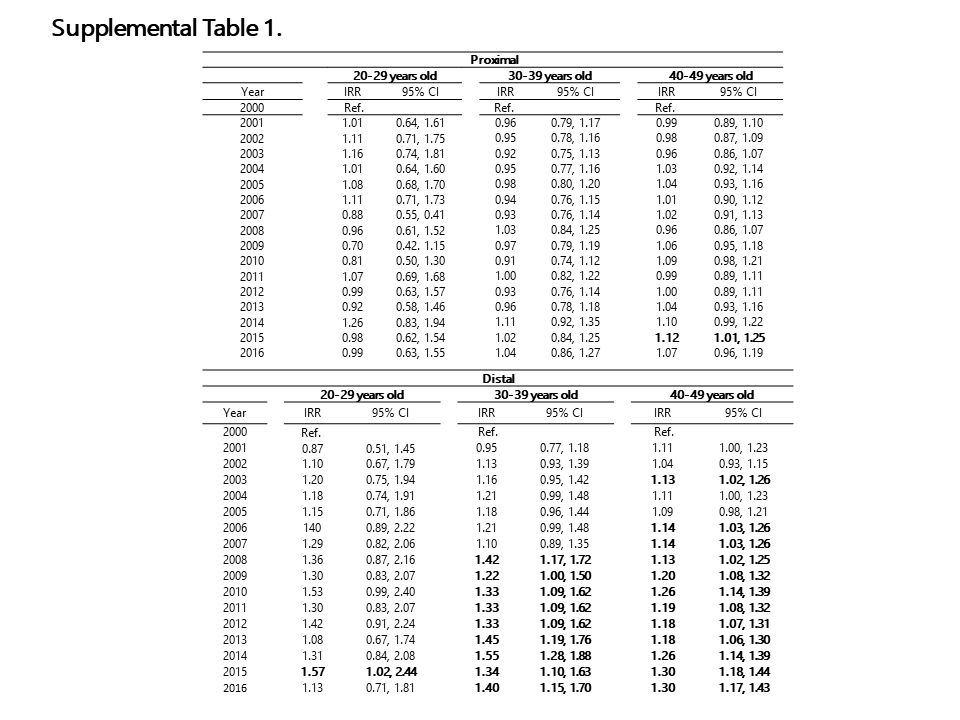

Supplement: Supplementary Materials — Incidence rate ratios (IRR) and 95% confidence intervals (95% CI) for proximal and distal colon cancers, stratified by age. [file 9841295.f1.tif]
